# The utility of delivery ward register data for determining the causes of perinatal mortality in one specialized and one general hospital in south Ethiopia

**DOI:** 10.1186/s12887-021-03058-4

**Published:** 2022-01-03

**Authors:** Tesfalidet Beyene, Catherine Chojenta, Roger Smith, Deborah Loxton

**Affiliations:** 1 Priority Research Center for Healthy Lungs, Faculty of Health and Medicine, University of Newcastle, Callaghan, NSW Australia; 2 Hunter Medical Research Institute, Lot 1 Kookaburra Circuit, New Lambton Heights, NSW 2305 Australia; 3 Centre for Women’s Health Research, Faculty of Health and Medicine, University of Newcastle, Callaghan, NSW Australia; 4 The Mothers and Babies Research Centre at the Hunter Medical Research Institute, University of Newcastle, Callaghan, NSW Australia

**Keywords:** Perinatal deaths, Birth, Utility, Delivery ward register, Ethiopia

## Abstract

**Background:**

Globally, the burden of perinatal mortality is high. Reliable measures of perinatal mortality are necessary for planning and assessing prenatal, obstetric, and newborn care services. However, accurate record-keeping is often a major challenge in low resource settings. In this study we aimed to assess the utility of delivery ward register data, captured at birth by healthcare providers, to determine causes of perinatal mortality in one specialized and one general hospital in south Ethiopia.

**Methods:**

Three years (2014–2016) of delivery register for 13,236 births were reviewed from July 12 to September 29, 2018, in two selected hospitals in south Ethiopia. Data were collected using a structured pretested data extraction form. Descriptive statistics assessed early neonatal mortality rate, stillbirth rate, perinatal mortality rate and causes of neonatal deaths. Factors associated with early neonatal deaths and stillbirths were examined using logistic regression. The adjusted odds ratios with a 95% confidence interval were reported to show the strength of the association.

**Result:**

The perinatal mortality ratio declined from 96.6 to 75.5 per 1000 births during the three-year study period. Early neonatal mortality and stillbirth rates were 29.3 per 1000 live births and 55.2 per 1000 total births, respectively. The leading causes of neonatal death were prematurity 47.5%, and asphyxia 20.7%. The cause of death for 15.6% of newborns was not recorded in the delivery registers. Similarly, the cause of neonatal morbidity was not recorded in 1.5% of the delivery registers. Treatment given for 94.5% of neonates were blank in the delivery registers, so it is unknown if the neonates received treatment or not. Factors associated with increased early neonatal deaths were maternal deaths and complications, vaginal births, APGAR scores less than 7 at five minutes and low birth weight (2500 g). Maternal deaths and complications and vaginal births were associated with increased stillbirths.

**Conclusion:**

Our findings show that an opportunity exists to identify perinatal death and newborn outcomes from the delivery ward registers, but some important neonatal outcomes were not recorded/missing. Efforts towards improving the medical record systems are needed. Furthermore, there is a need to improve maternal health during pregnancy and birth, especially neonatal care for those neonates who experienced low APGAR scores and birth weight to reduce the prevalence of perinatal deaths.

**Supplementary Information:**

The online version contains supplementary material available at 10.1186/s12887-021-03058-4.

## Background

Globally, the burden of perinatal mortality is high and intrapartum complications are important contributors to perinatal deaths especially in low-income countries [1–3]. Perinatal mortality is an important indicator of the quality of antenatal, intrapartum, and newborn care [4, 5]. Perinatal mortality refers to the number of stillbirths and deaths in the first week of life (early neonatal mortality). For every baby who dies in the first week after birth, another is born dead (stillbirths) [4]. For international comparison, the World Health Organization (WHO) defines stillbirth as a baby born with no signs of life at or after 28 weeks gestation [6]. Worldwide, 2.6 million stillbirths and 2.6 million neonatal deaths occur each year. Most of these deaths occur in low-middle income countries [7–9]. Sub-Saharan Africa and South Asia account for three-quarters of stillbirths. About 1.3 million (half) of all stillbirths occur during labour and birth [7]. Newborns face the highest risk of dying in their first four weeks of life, at an average global rate of 18 deaths per 1000 live births [9].

Perinatal death is associated with poor maternal health care, inadequate care during pregnancy and childbirth, inappropriate management of complications, poor hygiene during the first critical hours after birth and poor newborn care [4, 7, 10]. It has been reported that preterm birth, intrapartum complications (birth asphyxia), infection, and hypertensive disease are the most common contributors to perinatal death in low- and middle-income countries (LMIC) [9, 11–13]. Most perinatal deaths are preventable with the provision of high-quality care and timely interventions, especially during labour and delivery [13].

Globally there is a scarcity of quality information on the causes of perinatal death [14]. Reliable measures of perinatal mortality are necessary for planning and assessing prenatal, obstetric, and newborn care services. Accurate record-keeping is often a major challenge in low resource settings [5, 15]. Thus, the true burden of perinatal death is unclear. The WHO recommends collecting data on intrapartum care and neonatal mortality rates (NMR) as part of emergency obstetric care indicators. This will allow health care providers to take the most appropriate measures to prevent maternal and perinatal deaths [16]. However, in most countries, particularly where the estimated burden of perinatal death is highest, most stillbirths and half of all newborn deaths are not registered [14].

Ethiopia has achieved remarkable declines in under-five mortality rates [17, 18]. However, neonatal mortality rates remain unacceptably high [19]. A 2020 meta-analysis of perinatal mortality has shown that perinatal mortality in Ethiopia was 51.3 per 1000 total births (95% CI: 40.8–62.8) [20], and stillbirth and early neonatal mortality rates were 36.9 per 1000 births (95% CI: 27.3–47.8) and 29.5 per 1000 live births (95% CI: 23.9–35.6), respectively. This study has highlighted that the reduction trend of overall perinatal mortality was slow in the country.

Accurate recording and reporting of the number of stillbirths and neonatal deaths provide the data needed to improve and target health system responses and to measure outcomes in relation to policy and practice initiatives. Thus, we aimed to assess the utility of delivery ward register data, captured at birth by healthcare providers, to determine the causes of perinatal mortality in one specialized and one general hospital in south Ethiopia.

## Methods

### Study area

We collected secondary data from Hawassa University Comprehensive Specialized Hospital (HUCSH) and Durame General Hospital (DGH), Southern Nations, Nationalities and Peoples’ Region (SNNPR). The SNNPR is located in the south part of Ethiopia. The capital city (Hawassa) is located 285 km from Addis Ababa. The region has a total population of 15 million people, of whom 50% are women [21]. The 2014 Regional Health Bureau reported that the region has 21 public hospitals [22].

HUCSH is a referral teaching hospital, and top level ranked in the three-tier Ethiopian health care system. It delivers services for more than 18 million people. It has over 400 beds for inpatient services including three operating rooms for obstetric and gynaecological surgeries. Over 4000 births per year occur within the hospital, of which 30 to 35% are caesarean section. The DGH is located in Durame City and serves as a referral hospital for the Kembata Tembaro zone. The hospital provides services to over 1 million people and oversees 1700 deliveries annually. At the time of this study, each hospital has a neonatal intensive care unit that provides speciality care for newborns. However, the teaching hospital (HUCSH) is well equipped and has a higher number of paediatricians/neonatologists than the regional hospital.

### Study design and period

A hospital-based retrospective study was conducted to assess the utility of the delivery register data to determine the causes of perinatal death in both hospitals over three years (2014–2016). The study was carried out from 12 July to 29 September 2018.

### Study population

The delivery ward register of all deliveries between 2014–2016.

Documentation in delivery ward registers is routinely undertaken by facility health care providers and used to follow ward admissions and discharges after birth. Maternal age, mode of delivery, maternal wellbeing, newborn sex, birth outcomes, care and interventions for newborns are documented in this register.

### Inclusion and Exclusion criteria

#### Inclusion

Delivery registries, which have recorded details of birth and deaths of the newborns.

#### Exclusion criteria

Delivery before 28 weeks of gestation (abortion) had been excluded since they were not stillbirths or early neonatal death.

### Source of data and Data collection procedure

Data was collected using a structured pretested data extraction form. The extraction form was developed by examining the registration books of participating facilities. A template was prepared using an online survey tool *(*Survey Gizmo*)* and downloaded on iPads for offline data collection. Two data collectors and supervisors who had experience in data extraction were recruited to collect data. Delivery ward register data were reviewed, and the mothers’ cards were identified by medical registration/card number. Two data assistants were recruited to identify newborn charts. Data collected from delivery ward registers include maternal age, mode of delivery, APGAR score, birth weight, whether death occurred before or after arrival at the health facility, breastfeeding status, treatment given, treatment outcomes, and maternal complications. The principal investigator and supervisors oversaw the data collection process by checking the information collected and providing feedback.

### Quality assurance

Before actual data extraction, a pre-test was conducted to assess whether appropriate variables were included in the data extraction form. All required amendments to the tool were completed based on the pre-test. In addition to the pre-test, data collectors and supervisors were recruited based on prior experience in data extraction techniques. Two days of training was undertaken by data collectors and supervisors. Training focused on the study rationale, use of Ipads, Survey Gizmo, handling of data and the techniques for filling in the data extraction form.

### Data processing and analysis

Data were exported from Survey Gizmo into SPSS software (version 20) for analysis. Descriptive statistics were used to show early neonatal mortality rates (ENMR), stillbirth rates and perinatal mortality rates (PNMR). The PNMR was calculated by dividing the total number of neonatal deaths and stillbirths for the three-year period with the total amount of births within the same period and then converted to 1000 total births. The overall level of ENMR was calculated by dividing the total number of newborn deaths for the three-year period with the total number of live births within the same period and then translated into 1000 live births. The stillbirth rate was calculated by dividing the total number of stillbirths for the three-year period with the total number of births and then translated to 1000 total births. Trends in overall neonatal and perinatal mortality, as well as stillbirth rates during the period were examined using a simple linear regression model.

The univariable logistic regression model was used to obtain unadjusted associations between early neonatal deaths and stillbirth, and maternal and neonatal characteristics. All variables having p-values <0.2 were entered into a multivariable logistic regression model to control for possible confounding factors. Strength of association was expressed as adjusted odds ratio (AOR) with 95% confidence intervals (CI). The goodness of fit of the models was examined using the Hosmer-Lemeshow χ^2^ test with 10 subgroups, where p > 0.05 indicated a satisfactory model. Furthermore, variance inflation factor (VIF) was used to check multicollinearity among independent variables. However, the dichotomous measure of maternal complications (Yes/No) had multicollinearity with types of maternal complications, so we dropped types of complications from the multivariable models. Twin pregnancies were excluded from the analysis as they have high odds of death and may distort the effect size.

## Results

A total of 13,236 birth were included in this study. Of the total delivery register, 45% of records were from the year 2016. The majority of data (64.8%) were collected from Hawassa University comprehensive specialized hospital. Over one third (37.6%) of women were in the age category 21–25 years. Most (64.8%) women gave birth vaginally. Sixty-eight mothers died in hospitals and 15.7% of mothers developed complications. Pre-eclampsia or eclampsia was the leading (31.0%) maternal complication. Maternal complications in 16.0% of mothers were not specified/recorded in the delivery register. Four hundred and thirteen (3.1%) mothers had a history of previous caesarean sections. The majority (91.4%) of births were singleton (Table 1).

**Table 1:** Pregnancy outcomes and maternal characteristics in one specialized and one general hospital in south Ethiopia, 2014–2016

Variables	Number (%)
**Year, n = 13,236**	
2014	1564 (11.8)
2015	5633 (42.6)
2016	6039 (45.6)
**Name of the Hospital, n = 13,236**	
Durame Hospital	4655 (35.2)
Hawassa Referral Hospital	8581 (64.8)
**Age category, n = 13,236**	
<=20	2139 (16.2)
21–25	4983 (37.6)
26–30	4432 (33.5)
31–35	1315 (9.9)
>35	367 (2.8)
**Mode of delivery, n = 13,236**	
Spontaneous Vaginal delivery	7142 (54)
Caesarean section	4670 (35.2)
Episiotomy	105 (0.8)
Forceps or vacuum extraction	1319 [10]
**Maternal status, n = 13,236**	
Died	68 (0.5)
Stable	13,084 (98.9)
Unstable or deteriorated or referred to the next health facility	84 (0.6)
**Maternal complication, n = 13,236**	
Yes	2081 (15.7)
No	11,155 (84.3)
**Type of complication** ^**a**^**, n = 2081**	
Pre-eclampsia /Eclampsia	646 (31.0)
Antepartum haemorrhage	271 (13.0)
Postpartum Haemorrhage	164 (7.9)
Sepsis	36 (1.7)
Obstructed labor/Prolonged labor	459 (22.0.)
Uterine rupture	83 (4.0.)
Premature Rupture of the Membrane	89 (4.3)
Cephalopelvic disproportion	36 (1.7)
Complication referred	33 (1.6)
Other*	82 (3.9)
Not recorded or missing	334 (16.0)
**Previous history of caesarean section, n = 13,236**	
Yes	413 (3.1)
No	12,823 (96.9)
**Pregnancy outcome, n = 13,236**	
Single	12,096 (91.4)
Twin	388 (2.9)
Stillbirths	739 (5.6)
Macerated	13 (0.1)

Other* = Anaemia, Diabetic Mellitus, HIV, malaria, Hepatitis, Pneumonia, Pulmonary edema, bad obstetric history, cardiac disease, ^a^ more than one answer

### Characteristics of neonates

More than half (53.7%) of neonates were male. Nine percent of neonates had APGAR scores of less than seven at five minutes. Of the total neonates, 1622 (12.6%) were low birth weight (< 2500 g). One in nine (11.0%) neonates developed morbidity. The majority (79.8%) of the morbidities were related to prematurity, followed by perinatal asphyxia (12.2%). However, the cause of the newborn morbidity was not recorded for 22 (1.5%) newborns in the delivery register. Three hundred and seventy-seven (2.9%) neonates died. Many of the neonates died at the hospitals (93.9%). Breastfeeding was initiated within one hour in 94.6% of neonates. However, mode of feeding was not recorded for 41(0.3%) newborns. Treatment given in 94.5% of neonates were blank in the delivery register, so it is unknown if neonates received treatment or not (Table 2).

**Table 2:** Neonatal characteristics of neonates delivered in one specialized and one general hospital in south Ethiopia, 2014–2016

Variables	Number (%)
**Sex for live births, n = 12,872**	
Female	5957 (46.3)
Male	6915 (53.7)
**APGAR Score at fifth minutes, n = 12,872**	
<7	1139 (8.8)
> = 7	11,733 (91.2)
**Birth weight (gm), n = 12,872**	
<2500	1622 (12.6)
> = 2500	11,250 (87.4)
**The Newborn developed morbidity, n = 12,872**	
Yes	1420 (11.0)
No	11,452 (89)
**Newborn morbidity** ^**a**^**, n =** 1420	
Prematurity	1134 (79.8)
Perinatal Asphyxia	173 (12.1)
Sepsis	63 (4.4)
Congenital malformation	21 (1.5)
Meconium Aspiration	8 (0.6)
HIV	24 (1.7)
Other	43(3.0.)
Not recorded or missing	22 (1.5)
**Newborn outcome, n = 12,872**	
Died	377 (2.9)
Not died	12,495 (97.1)
**Survived newborn n = 12,495**	
Home and improved	12,185 (97.5)
Referred	310 (2.5)
**Newborn died n = 377**	
Live birth died after arrival or delivery in facility	354 (93.9)
Live birth died before arrival at facility	23 (6.1)
**Mode of feeding, n = 12,872**	
Breast feeding initiated after one hour	383 (3.0)
Breastfeeding initiated within one hour	12,182 (94.6)
Other feeding not recorded	266 (2.1)
Not recorded	41 (0.3)
**Treatment given** ^**a**^**, n = 12,872**	
Oxygen/Resuscitation	488 (3.8)
Kangaroo Mother Care	153 (1.2)
Antibiotics	83 (0.6)
Blood transfusion	13 (0.1)
Other	101 (0.8)
Not recorded	12,166 (94.5)

^a^ more than one # the n includes live twin births

### Cause of early neonatal death

**Fig. 1:**
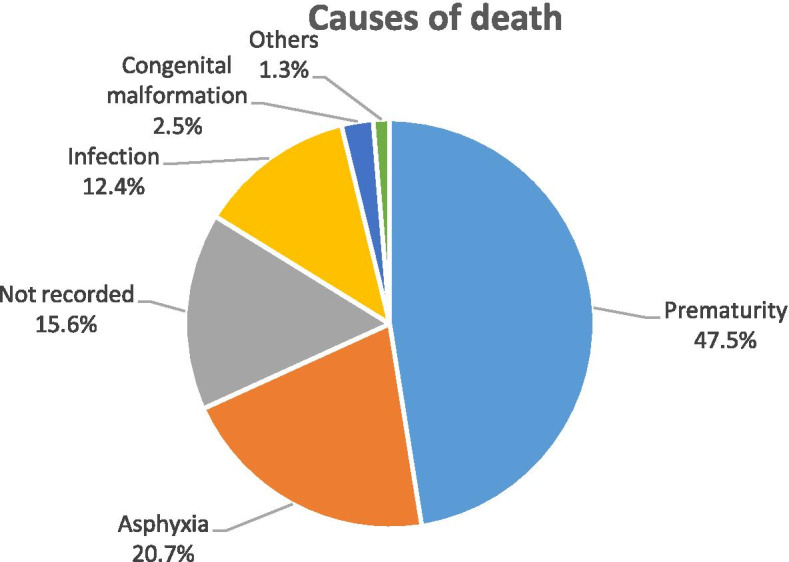
Cause of early neonatal deaths in one specialized and one general hospital in south Ethiopia, 2014–2016

The leading causes of neonatal death were prematurity 179 (47.5%), followed by asphyxia 78 (20.7%). The cause of death for 59 (15.6%) newborns were not recorded in the delivery register (Fig. 1).

### Perinatal death

**Fig. 2:**
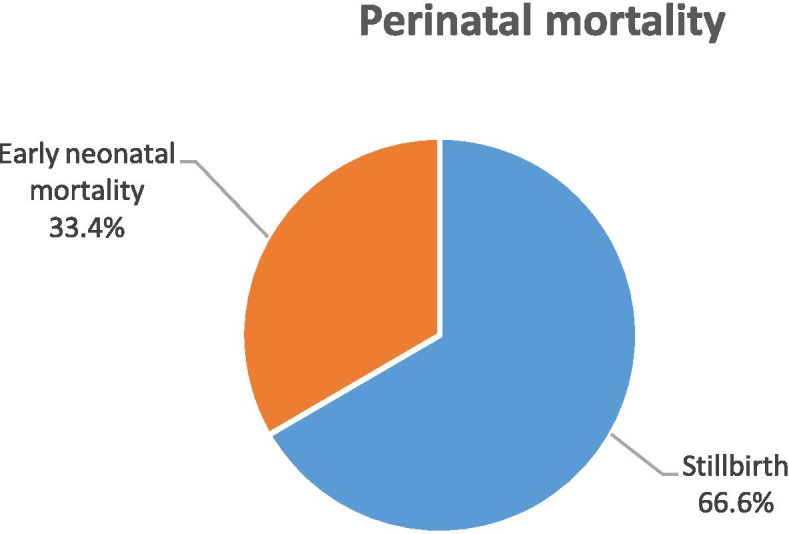
Percentage of neonatal deaths and stillbirths in one specialized and one general hospital in south Ethiopia, 2014–2016

A total of 1129 perinatal deaths were identified during the study period. The PNMR in the study was 82.9 per 1000 total births (95% CI 78.3, 87.5). Two thirds (66.6%) were stillbirths and 33.4% were neonatal deaths (Fig. 2).

The number of perinatal deaths (early neonatal death and stillbirth) per year and the corresponding PMRs with 95% confidence intervals are presented in Table 3. The overall PMR was 82.9 (95%CI; 78.3–87.6). The overall stillbirth and early neonatal mortality rates were 55.2 (95%CI; 51.4–59.2) per 1000 total births and 29.3 (95%CI; 26.4–32.4) per 1000 live births, respectively.

**Table 3:** Annual perinatal mortality ratios over the three years period (2014–2016) in one specialized and one general hospital in south Ethiopia

Year	Number of deliveries	Number of live births	Number of stillbirths	Stillbirths per 1000 deliveries with 95%CI	Number of early neonatal deaths	NMR per 1000 live births with 95%CI	PNMR with 95%CI
2014	1615	1538	77	47.7 (37.3–58.1)	79	51.4 (40–62.4)	96.6 (82.2–110.1)
2015	5787	5454	333	57.5 (51.5–63.5)	169	30.9 (26.3–35.5)	86.7 (79.5–93.9)
2016	6222	5880	342	55 (49.3–60.7)	129	21.9 (18.2–25.6)	75.5 (69.1–82.3)
Total	13,624	12,872	752	55.2 (51.4–59.2)	377	29.3 (26.4–32.4)	82.9 (78.3–87.6)

# the n includes live twin births

### Trends of perinatal deaths

**Fig. 3:**
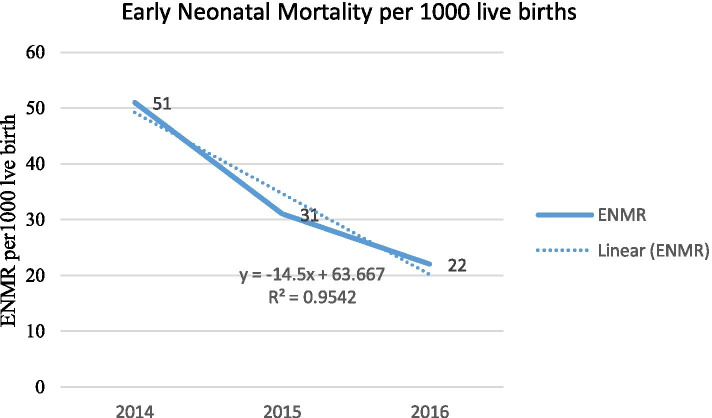
Trends in early neonatal mortality rate in one specialized and one general hospital in south Ethiopia, 2014–2016. R^2^ refers to a measure of the reliability of the relation between the years and early neonatal deaths

Across the study period, the trend of the ENMR varied with the highest observed in 2014 with 51.4 per 1000 live births and the lowest observed in 2016 with 21.9 per 1000 live births. There was a slow declining trend in ENMR during the reference period in the study area with a gradient of 14.5 on a linear scale (Fig. 3).

**Fig. 4:**
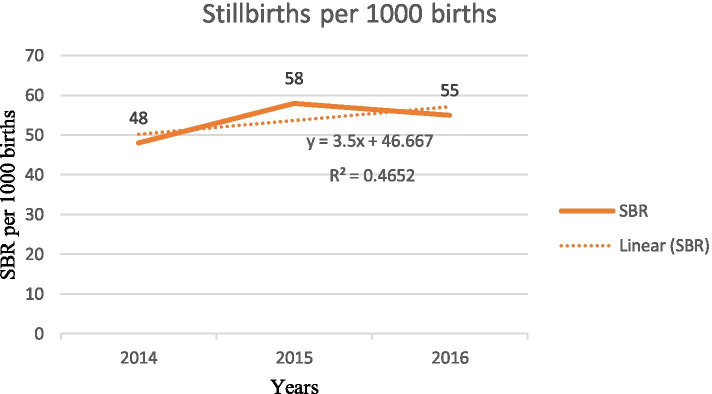
Trends in stillbirth rates in one specialized and one general hospital in south Ethiopia, 2014–2016. R^2^ refers to a measure of the reliability of the relation between the years and early stillbirths

Across study years, the trend of stillbirths varied, with the highest being observed in 2015 with 57.5 per 1000 births and the lowest observed in 2014 with 47.7 per 1000 births. There was an increasing trend in stillbirths during the reference period in the study area, with a gradient of 3.5 on a linear scale (Fig. 4).

**Fig. 5:**
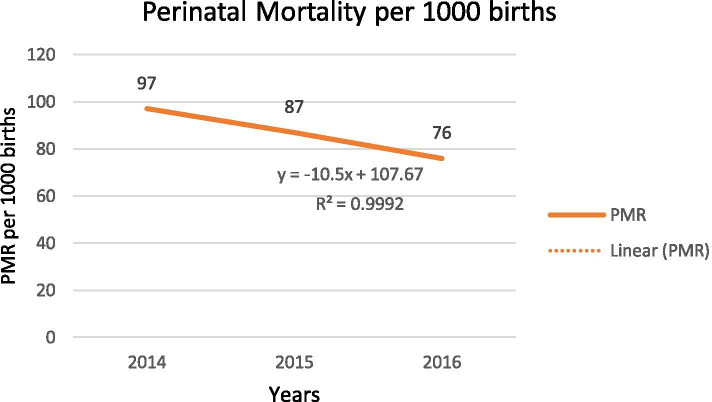
Trends in perinatal mortality ratio in one specialized and one general hospital in south Ethiopia, 2014–2016. R^2^ refers to a measure of the reliability of the relation between the years and perinatal mortality

Across the study period the trend of the PMRs varied, with the highest being observed in 2014 with 96.6 per 1000 births and the lowest observed in 2016 with 75.5 per 1000 births. There was a reduction in trend in PMRs during the reference period in the study area, with a gradient of 10.5 on a linear scale (Fig. 5).

### Maternal complications in perinatal deaths (early neonatal deaths and stillbirths)

**Fig. 6:**
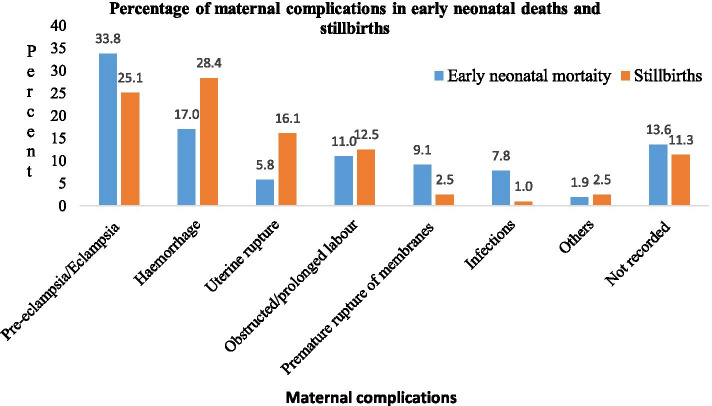
The percentages of maternal complications in perinatal mortality in one specialized and one general hospital in south Ethiopia, 2014–2016

The leading cause of early neonatal deaths relating to maternal complications was pre-eclampsia or eclampsia (33.8%) followed by haemorrhage (17.0%). However, the cause of early neonatal deaths in 13.6% of births related to maternal complications was not recorded in the delivery register. The leading cause of stillbirths due to maternal complications was haemorrhage (28.4%) followed by pre-eclampsia/eclampsia (25.1%). However, the cause of stillbirths in 11.3% of delivery’s related to maternal complications was not recorded in the delivery register (Fig. 6).

### Determinants of early neonatal mortality

Regarding univariable analysis, age of the mother, mode of delivery, maternal death and complications, history of caesarean section, sex of the newborn, APGAR score less than 7 at five minutes and low birth weight (<2500 g) were associated with early neonatal deaths. In multivariable analysis, neonates born to mothers who died/unstable (AOR 7.54; 95% CI 4.14–13.71) and mothers who had complications (AOR 2.71; 95% CI 2.01–3.66) had higher odds of death than neonates born to mothers who survived and didn’t experience complications. Vaginal birth was found to increase the likelihood of early neonatal deaths compared to caesarean section (AOR 3.08; 95% CI 2.23–4.25). Neonates who had an APGAR score of less than 7 at five minutes (AOR 4.40; 95% CI 3.28–5.91) were found to have an increased likelihood of neonatal death than neonates who had APGAR sore 7 or more at five minutes. Neonates who had low birth weight (< 2500 g) (AOR 6.4; 95% CI 4.77–8.58) had a higher odds of death than neonates who had birth weight 2500 g or more (Table 4).

**Table 4:** Determinants of early neonatal mortality in one specialized and one general hospital in south Ethiopia, 2014–2016

Variables	Early neonatal deaths			
	Died (n = 318)	Survived (n = 11,778)	Crude OR (95%CI)	Adjusted OR (95%CI)
**Age category**				
<=20	45 (14.1)	1946 (16.5)	1	
21–25	106 (33.3)	4495 (38.1)	1.02 (0.72–1.45)	1.25 (0.84–1.86)
26–30	108 (34.0)	3929 (33.4)	1.19 (0.84–1.69)	1.27 (0.85–1.89)
31–35	47 (14.8)	1112 (9.4)	1.82 (1.21–2.76)	1.78 (0.98–2.60)
>35	12 (3.8)	296 (2.5)	1.75 (0.92–3.35)	1.32 (0.63–2.78)
**Mode of delivery**				
Vaginal delivery	255 (80.2)	4170 (35.4)	2.22 (1.67–2.93)	3.08 (2.23–4.25)
Caesarean section	63 (19.8)	7608 (64.6)	1	1
**Maternal status**				
Died/unstable or deteriorated	44 (13.8)	52 (0.44)	36.21 (23.81–55.05)	7.54 (4.14–13.71)
Stable	274 (86.2)	11,726 (99.5)	1	1
**Maternal complication**				
Yes	155 (48.7)	1450 (12.3)	6.77 (5.40–8.50)	2.71 (2.01–3.66)
No	163 (51.3)	10,328 (87.7)	1	1
**Previous history of caesarean section**				
Yes	2 (0.6)	391 (3.3)	0.18 (0.04–0.74)	0.35 (0.07–1.65)
No	316 (99.4)	11,387 (96.7)	1	1
**Sex**				
Female	110 (34.6)	5474 (46.5)	1	1
Male	208 (65.4)	6304 (53.5)	1.64 (1.29–2.07)	1.51 (0.95–1.96)
**APGAR Score at fifth minutes**				
<7	165 (51.9)	715 (6.1)	16.68 (13.22–21.05)	4.40 (3.28–5.91)
> = 7	153 (48.1)	11,063 (93.9)	1	1
**Birth weight (gm)**				
<2500	202 (63.5)	1031 (8.7)	18.15 (14.32–23.01)	6.4 (4.77–8.58)
> = 2500	116 (36.5)	10,747 (91.3)	1	1

# n does not include twin births

### Determinants of stillbirths

Univariable age of the mothers, mode of delivery, maternal deaths and complications were associated with increased stillbirths. In multivariable analysis, the odds of having stillbirths was two times higher (AOR 2.47; 95% CI 2.06–2.96) among mothers who had vaginal births compared to caesarean births. Mothers who died/unstable (AOR 2.51; 95% CI 1.74–3.62) and experienced complications (AOR 8.59; 95% CI 7.26–10.17) had a higher odd of having stillbirths compared to mothers who survived and didn’t have complications (Table 5).

**Table 5:** Determinants of stillbirths in one specialized and one general hospital in south Ethiopia, 2014–2016

Variables	Stillbirths			
	Fetal deaths (n = 752)	Liveborn neonates (n = 12,096)	Crude odds ratios	Adjusted odds ratio with 95%CI
**Age category**				
<=20	103 (13.7)	1991 (16.5)	1	1
21–25	241 (32.1)	4601 (38.0)	1.01 (0.80–1.28)	1.07 (0.84–1.37)
26–30	252 (33.5)	4037 (33.4)	1.21 (0.95–1.53)	1.22 (0.96–1.56)
31–35	111 (14.8)	1159 (9.6)	1.85 (1.40–2.44)	1.65 (0.98–2.21)
>35	45 (5.9)	308 (2.5)	2.82 (1.95–4.09)	2.60 (0.75–3.86)
**Mode of delivery**				
Vaginal delivery	541 (71.9)	7863 (65.0)	1.38 (1.17–1.62)	2.47 (2.06–2.96)
Caesarean section	211 (28.1)	4233 (35.0)	1	1
**Maternal status**				
Died/unstable	54 (7.2)	96 (0.8)	9.6 (6.86–13.61)	2.51 (1.74–3.62)
Stable	698 (92.8)	12,000 (99.2)	1	1
**Maternal complication**				
Yes	398 (52.9)	1605 (13.3)	7.34 (6.30–8.56)	8.59 (7.26–10.17)
No	354 (47.1)	10,491 (86.7)	1	1

# n does not include twin births

## Discussion

To our knowledge, this study is the first of its kind to be undertaken in Ethiopia that utilizes delivery ward register data to identify perinatal deaths and assess gaps in documentation/recording of newborn outcomes. The findings of our study show that perinatal deaths declined over the three-year period in the study area. Our findings also show that an opportunity exists to identify perinatal death and newborn outcomes from the delivery registers, but some important neonatal outcomes were either not recorded or not recorded reliably.

We found that maternal deaths and complications, vaginal births, newborn APGAR score less than 7 at five minutes and newborn birth weight (<2500 g) were associated with increased early neonatal deaths. Our findings also show that vaginal births, maternal deaths and complications were associated with an increased likelihood of stillbirths.

The findings of this study identified several limitations in the maintenance of records in both hospitals. One of our findings revealed a lack of a universal system for recording births and care provided to newborns in both hospitals. The cause of neonatal death was not recorded in nearly one-sixth of the delivery register. Similarly, the cause for neonatal morbidity was not recorded in 1.5% of the delivery register. A study undertaken in Tanzania found that 33.3% of all newborn deaths were properly documented but the cause of death was only reported in 38% of cases [23]. A study in Bangladesh revealed that the quality of neonate medical records by cause of death was lower when compared with adult records [24]. This suggests a gap in low resource settings in recording the cause of newborn deaths and that information provided within such records was often inadequate. A meta-analysis on perinatal death reviews done in low and middle-income countries revealed reviewing deaths may reduce perinatal death by 30% and improve quality of care [25]. The findings of the present study imply there is a gap in records of the delivery register that needs to be improved to identify causes of newborn deaths.

In our study, 94.5% of treatments neonates received were not recorded in the delivery register indicating poor documentation and a missed opportunity for identifying the efficacy of treatment outcomes. It has been reported that poor documentation of medical records leads to suboptimal care [26]. A study in Malawi demonstrated the importance of record-keeping in assessing critically ill neonates and determining factors that were contributing to newborn deaths [27]. Taken together, these studies suggest that poor documentation may result in suboptimal care and hinder treatments to reduce newborn deaths.

Studies have indicated that most perinatal deaths occur due to maternal complications [2, 28]. In the current study, preeclampsia or eclampsia and haemorrhage were the leading causes of early neonatal deaths and stillbirths, respectively. However, 13.6% of causes of early neonatal deaths and 11.3% of stillbirths related to maternal complications were not recorded in the delivery register. Maternal complications were often missing from the delivery register. These data indicate a need for improvement of documentation in delivery registers to facilitate preventive measures relating to maternal complications and perinatal deaths.

Our findings indicate that the trend of newborn mortality varied over the three years. Newborn mortality reduced from 51 to 22 per 1000 live births. While the reduction in child mortality rates in Ethiopia are encouraging [19, 29] this reduction is likely to be unchanged if the quality of documentation in delivery registers and newborn care is not addressed. A study undertaken in Ethiopia showed that the neonatal mortality rate was reduced by 1.9% annually [30]. The reason for the reduction in neonatal mortality can be explained by government commitments to increase (i) service availability to the mother and newborn and (ii) increasing the capacity of neonatal intensive care in Ethiopia. The reduction coincides with the deployment of a large number of health extension workers in the community to create awareness on the use of maternal health care services. Such improvements in outcomes related to policy changes can only be measured if accurate data are collected.

This study showed stillbirth rates of 55.2 per 1000 total births over the three-year study period. This prevalence was lower than other community and facility-based studies done in Ethiopia, at 71 and 85 per 1000 total births respectively [31, 32]. The reason for the difference can be explained by variation in the study period (the latter studies collected data from 2011 to 2013); every year there is an improvement in maternal health care service utilization and training of health care providers in Ethiopia. In this study, the trend of stillbirth rates has increased over the three years. Stillbirth rates increased by 14.6%, with the highest stillbirth rates in 2015; an increase of 20.8%. A possible explanation for this is not clear, but it may be due to a high number of complicated pregnancy referrals from other facilities. Another reason may be the fact that in this study the highest maternal death was reported in 2015 as a multicounty survey on maternal and newborn health done in low- and middle-income countries revealed that the majority of stillbirths occur in mothers with medical or obstetric complications [2].

The association between maternal deaths and complications and perinatal deaths have been reported in multiple studies [33–35]. Our findings are consistent with other findings in Ethiopia which showed both maternal deaths and complications increase the risk of perinatal deaths [36–39]. Taken together, these studies suggest the need to improve maternal health during pregnancy and birth to prevent perinatal deaths.

Our study found that vaginal births increase the risk of perinatal deaths. A multicounty survey study investigating maternal and newborn health report a higher prevalence of vaginal births in pregnancies resulting in perinatal deaths [40]. A case-control study in Ethiopia found that perinatal deaths were less likely among mothers who delivered by caesarean section than vaginal births [41]. In contrast, a cross-sectional study of perinatal deaths in Southern Ethiopia revealed that vaginal delivery reduced the risk of perinatal deaths [40]. A possible explanation for the differences in findings may result from the differences in sample size, data collection methods and study period.

Our results showed that low APGAR scores at five minutes and low birth weight increased the likelihood of early neonatal deaths. This is consistent with previous research, which reports low APGAR scores and birth weight were significantly associated with increased risk of early neonatal deaths [37, 41–43]. Furthermore, a study of predictive factors of low APGAR score at five minutes among singleton births in northwest Ethiopia has shown that low birth weight is significantly associated with increased low APGAR scores at five minutes [44]. The study in northwest Ethiopia indicates the effect of low birth weight on APGAR scores which in turn may increase the risk of early neonatal deaths [44]. These findings underscore the need for improved neonatal care for newborns who have low APGAR scores and birth weight to reduce the risk of neonatal death.

### Limitation of the study

This study has three limitations. Firstly, the collected data were secondary, therefore it was difficult to collect all required information to assess predictors and causes of perinatal deaths. Secondly, the study was conducted in two hospitals only, thus the findings may not reflect the situations at all health facilities in the country. Thirdly, data were collected from the hospital delivery ward register and therefore, newborn deaths occurring after discharge may have been missed.

## Conclusion

Though the magnitude of perinatal deaths declined by 21.6% over the three years, there is a wide range of problems with data collected in delivery ward registers. This study has shown a deficiency in data records, with the cause of death frequently not documented accurately or not recorded in delivery registers. Our findings show there is an opportunity to identify perinatal death and newborn outcomes from the delivery registers, but some important neonatal outcomes were not recorded. Appropriate documentation of the delivery register could have a major health implication, not only for newborn and maternal outcomes but also for program planning and evaluation. Training of health care providers is warranted to create and increase awareness of the importance of delivery registries and medical records to identify causes of the perinatal deaths occurring in health facilities. Policymakers and stakeholders should revise medical record forms and develop clear guidelines consisting of important variables related to perinatal outcomes. Moreover, continuous supervision and assessment of delivery ward registers is necessary. There is also a need to improve maternal health during pregnancy and birth, especially neonatal care for those neonates who experienced low APGAR scores and birth weight to reduce the prevalence of perinatal deaths.

### Future research

Qualitative research is needed to increase understanding surrounding the possible reasons for incomplete records from the health care provider’s perspective. Furthermore, research nationwide, including lower level and higher-level health facilities is necessary.

### Implications of the study

This study showed that determining the cause of perinatal deaths using delivery ward registers was possible, but there are gaps in record-keeping resulting in incomplete or unreliable data. Accurate recording and reporting of reliable data including number and cause of death within hospitals are important for program planning and monitoring. The use of a delivery ward register is one method to enhance awareness of why newborns die by revealing what type of treatment was given or not given to the newborn and also provides the leading cause of death. This provides information to policymakers on what actions need to be taken to reduce preventable perinatal deaths. Furthermore, understanding treatment provision in relation to mortality helps to identify unmet clinical needs in the hospital. Thus, health managers and policymakers in the country should work on improving documentation systems.

## Supplementary Information

**Additional file 1.**

## Data Availability

The datasets used and/ or analysed during the current study are available from the corresponding author on reasonable request.
