# Supplementary material for: The utility of delivery ward register data for determining the causes of perinatal mortality in one specialized and one general hospital in south Ethiopia
Source: BMC Pediatr. 2022 Jan 3;22:6. doi: 10.1186/s12887-021-03058-4 (PMC8721979; doi:10.1186/s12887-021-03058-4)
Supplement: Supplementary file 1 — Additional file 1. [file 12887_2021_3058_MOESM1_ESM.docx]

**Retrospective medical record review to identify the causes of perinatal deaths in south Ethiopia**

**Instruction**: For the questions that have alternatives, circle to the response of the mother. Write appropriate response(s) on the space provided for questions for which alternatives are not given.

**Data extraction form for neonatal mortality**

**Name of the hospital**: ___________________

| S.No | Question | Response | Skipping |
| --- | --- | --- | --- |
| 102 | Delivery year | 1. 2014 2. 2015 3. 2016 |  |
| 1 | Mother age | ___________ Years |  |
| 2 | Delivery type | 1. Spontaneous vaginal delivery 2. Caesarean section 3. Forceps or vacuum extraction 4. Episiotomy 5. Other procedure (specify) |  |
| 3 | Previous caesarean section | 1. Yes 2. No |  |
| 3 | Maternal status | 1. Stable 2. Unstable /deteriorated and referred to the next health facility 3. Died |  |
| 4 | Obstetric complication | 1. Pre-eclampsia /Eclampsia 2. Antepartum hemorrhage 3. Postpartum hemorrhage 4. Sepsis 5. Obstructed/prolonged labour 6. Uterine rupture 7. PROM 8. Other obstetric complication _____________ 9. Complication referred |  |
| 5 | Newborn birth outcome | 1. Alive 2. Apgar score 5’__________________ 3. Sex___________ 4. Weight in grams ____________ 5. Still birth 6. Macerated 7. Live birth, died before arrival at facility 8. Live birth died after arrival or delivery in facility |  |
| 6 | Problem identified/Newborn morbidity | 1. Prematurity 2. Sepsis 3. Perinatal asphyxia 4. Congenital malformation 5. Other(specify)_________ |  |
| 7 | Breast feeding identified | 1. Brest feeding within 1 hour 2. Breast feeding initiated after 1 hour 3. Other feeding |  |
| 8 | Treatment given for newborn | 1. Oxygen/Resuscitation 2. Kangaroo Mother care 3. Antibiotics 4. Blood transfusion 5. Other(specify) |  |
| 9 | Treatment outcome | 1. Improved 2. Died 3. Referred 4. Other(specify)_____________ |  |
| 10 | Cause of death | 1. Prematurity 2. Infection 3. Asphyxia 4. Congenital malformation 5. Other(specify)_______________ |  |
